# Differential Requirement for CCR4 in the Maintenance but Not Establishment of the Invariant Vγ5^+^ Dendritic Epidermal T-Cell Pool

**DOI:** 10.1371/journal.pone.0074019

**Published:** 2013-09-12

**Authors:** Kyoko Nakamura, Andrea J. White, Sonia M. Parnell, Peter J. Lane, Eric J. Jenkinson, William E. Jenkinson, Graham Anderson

**Affiliations:** Medical Research Council Centre for Immune Regulation, University of Birmingham, Birmingham, United Kingdom; Oklahoma Medical Research Foundation, United States of America

## Abstract

Thymocytes expressing the invariant Vγ5 γδT-cell receptor represent progenitors of dendritic epidermal T-cells (DETC) that play an important immune surveillance role in the skin. In contrast to the bulk of αβT-cell development, Vγ5^+^ DETC progenitor development occurs exclusively in fetal thymus. Whilst αβT-cell development is known to require chemokine receptor mediated migration through distinct thymus regions, culminating in medullary entry and thymic egress, the importance and control of intrathymic migration for DETC progenitors is unclear. We recently revealed a link between Vγ5^+^ DETC progenitor development and medullary thymic epithelial cells expressing Aire, a known regulator of thymic chemokine expression, demonstrating that normal Vγ5^+^ DETC progenitor development requires regulated intramedullary positioning. Here we investigate the role of chemokines and their receptors during intrathymic Vγ5^+^ DETC progenitor development and establishment of the DETC pool in the skin. We report that thymic medullary accumulation of Vγ5^+^ DETC progenitors is a G-protein coupled receptor dependent process. However, this process occurs independently of Aire’s influences on intrathymic chemokines, and in the absence of CCR4 and CCR7 expression by DETC progenitors. In contrast, analysis of epidermal γδT-cells at neonatal and adult stages in CCR4^−/−^ mice reveals that reduced numbers of DETC in adult epidermis are not a consequence of diminished intrathymic embryonic development, nor deficiencies in initial epidermal seeding in the neonate. Collectively, our data reveal differences in the chemokine receptor requirements for intrathymic migration of αβ and invariant γδT-cells, and highlight a differential role for CCR4 in the maintenance, but not initial seeding, of DETC in the epidermis.

## Introduction

During the postnatal and adult periods, most T-cells produced in the thymus express the αβ form of T-cell receptor (αβTCR) complex, and are generated via a process involving random recombination at the *Tcra* and *Tcrb* gene loci to generate a pool of immature αβTCR^+^ thymocytes with a wide range of antigen specificities [1]. Such cells are then required to undergo stringent selection events based upon their ability to recognize self-peptide/MHC ligands expressed by thymic epithelial cells and dendritic cells. In contrast, during embryonic stages the first T-cells to be produced in the thymus are defined by expression of the γδTCR [2], [3]. γδT-cell development at these stages involves the sequential production of distinct waves of T-cells, each of which is defined by expression of an invariant γδTCR and a particular tissue tropism. Thus, thymocytes expressing the Vγ5/δV1 γδTCR initially appear around E14 of gestation [4], and represent the thymic progenitors of Vγ5^+^TCR Dendritic Epidermal T-cells, which represent an intraepithelial lymphocyte population linked to immune surveillance in the skin [5].

The generation of αβT-cells within established cortical and medullary microenvironments in the adult thymus is linked to an ordered process of intrathymic migration in which chemokines and their receptors play a key role. Several chemokine receptors demonstrate dynamic expression patterns during αβT-cell development including CXCR4/CCR7/CCR9, all of which have been linked to thymus entry and early T-cell progenitor development [6]–[12]. Significantly, migration of positively selected thymocytes from the cortex to the medulla, a process essential for αβT-cell tolerance induction, requires CCR7-mediated migration promoted by expression of CCL19/CCL21 by medullary stromal cells [13], with CCR7 also linked to thymic egress of newly selected T-cells [14], at least in the neonatal period. Interestingly, intrathymic expression of some chemokines are either absent (XCL1), reduced (CCL17, CCL19, CCL21, CCL22) or increased (CCL25) in the absence of Aire, a gene expressed by mTEC that also plays a key role in regulating availability of Tissue Restricted Antigens for αβT-cell tolerance induction [15], [16].

In contrast to αβT-cells, the potential importance of intrathymic migration through distinct thymus microenvironments for γδT-cell development, and the role of particular chemokines in this process, is not clear. Interestingly however, Vγ5^+^ DETC thymocyte progenitors are physically clustered with mTEC, including those expressing Aire [17], which correlates with the requirement for mTEC in Vγ5^+^ DETC progenitor maturation via their expression of Skint-1, a key regulator of DETC development [18]. Moreover, the induction of Aire^+^ mTEC development occurring as a result of RANKL expression on Vγ5^+^ DETC thymocyte progenitors demonstrates a reciprocal interaction between DETC progenitors and Aire^+^ mTEC. Importantly, however, the effect of altered chemokine expression caused by Aire deficiency on intrathymic Vγ5^+^ DETC progenitor migration is not clear. Indeed, while other studies reported a role for CCR4, whose ligands are altered by Aire deficiency [15], in the formation of a normal DETC in the epidermis of adult mice [19], the role of CCR4 during intrathymic Vγ5^+^ DETC progenitor migration and development, culminating in the initial seeding of the epidermis in the neonate, has not been fully studied.

Here, we have analysed the role of CCR4 and CCR7, both of which represent receptors for medullary chemokines [13], [20]–[22] and are shown here to be expressed by Vγ5^+^ DETC progenitors, in relation to the intrathymic migration and development of Vγ5^+^ T-cells. We show that Vγ5^+^ DETC progenitor localization to the thymic medulla, analogous to that shown previously for developing αβT-cells [23], is inhibited by pertussis toxin treatment, suggestive of a role for G-protein coupled chemokine receptors in this process. While Aire-mediated effects on chemokine expression does not alter Vγ5^+^ DETC progenitor localization in the thymic medulla, a process also unaltered in CCR4^−/−^ and CCR7^−/−^ embryonic thymus, we show that the diminished numbers of DETC selectively observed in the epidermis of adult CCR4^−/−^ mice is not a consequence of alterations in intrathymic T-cell development and initial seeding of the neonatal epidermis. Collectively, our data help redefine the role of CCR4 in DETC thymus development and skin homing.

## Materials and Methods

### Mice

Wild type C57BL/6, Aire^−/−^ [24], CCR4^−/−^ mice [25], and CCR7^−/−^ mice [26] were bred and maintained at the University of Birmingham Biomedical Services Unit. Indicated control mice are C57BL/6, mated alongside the indicated knockout strain. All animal work was performed in accordance with UK Home Office regulations and approved by the University of Birmingham Ethical Review Committee. For the generation of timed mated pregnancies, the day of detection of a vaginal plug was counted as day 0. Cell counts were obtained from embryos of known genotype using AccuCount Blank Particles according to manufacturers instructions (Spherotech), and data shown are indicative of the number of cells per embryo, unless otherwise indicated.

### Antibodies, Flow Cytometry and Cell Sorting

The following antibodies were used (all eBioscience unless otherwise stated): anti-TCRVγ5 (536, BD Biosciences), Anti-CD3 (145-2C11), Anti-CD45RB (C363.16A), anti-CCR4 (2G12, Biolegend), anti-pan CD45 (clone 30-F11). For CCR7 detection, cells were stained with mouse recombinant CCL19-Fc then stained with biotin conjugated anti-human IgG Fc gamma followed by fluorescent conjugated streptavidin. For apoptosis analysis, Annexin V (Annexin V-Biotin) apoptosis detection kit I (BD Biosciences) was used in accordance of manufacture’s instruction. Multicolour flow cytometry was performed with a BD-LSR Fortessa cell analyzer running FACSDIVA 6.2 software (BD Biosciences). Flow cytometry data analysis was performed using Flowjo software (Treestar). Cell sorting was performed with MoFlo XDP (Beckman Coulter) and Summit software (Dako).

### Confocal Microscopy

The following reagents were used for confocal microscopy: anti-medullary epithelium (monoclonal rat IgM antibody, clone ER-TR5, kind gift from W. van Ewijk) [27], anti-EpCAM1 (monoclonal rat IgG antibody, clone G8.8, kind gift from A. Farr) [28] conjugated to Alexa Fluor 647 (Invitrogen) and anti-CD8β biotin (YTS156.7.7, Biolegend), detected by Alexa Fluor 555 conjugated streptavidin (Invitrogen). FITC conjugated anti-TCR Vγ5 antibodies were amplified using FITC-Alexa Fluor 488 (Invitrogen) followed by Alexa Fluor 488 conjugated donkey anti-rat rabbit IgG (Invitrogen). ER-TR5 antibody was detected with Alexa Fluor 594 conjugated goat anti-rat IgM (Invitrogen). Tissues were embedded in OTC compound (Sakura Finetek) and frozen on dry ice. Frozen tissues were sliced into 6 µm thick sections by cryostat and fixed in acetone for 20 minutes. Confocal images were acquired using a LSM 510 Meta microscope (Zeiss) and analyzed using Zeiss LSM software. For quantitation of Vγ5^+^ cells in medulla, Vγ5^+^ cells were counted manually and the number was divided by the area of medulla, calculated using Zeiss LSM software, which was defined by CD8β negative and EpCAM positive area.

### Fetal Thymus Organ Culture and Pertussis Toxin Treatment

Freshly isolated E15 fetal thymus lobes were treated with 250 ng/ml Pertussis Toxin (Sigma) for 30 minutes at 37°C in Dulbecco’s Modified Eagles Medium supplemented with 10% FCS, 10 mM Hepes (Sigma), non-essential amino acids (Sigma), 50 µM 2-mercaptoethanol (Sigma), 100 IU/ml Penicillin and Streptomycin (Sigma), 4 mM Glutamine (Sigma). Thymus lobes were washed with Phosphate buffered saline (PBS) and placed in organ culture as described [29].

### Preparation of Epidermal And Dermal Cells For Flow Cytometry

For neonatal DETC analysis, day 0 neonatal back and belly skin was peeled off. For adult DETC analysis, ears were split into dorsal and ventral sides. Isolated skin was submerged in 20 mM EDTA PBS with the epidermal side down. After the incubation for 2 hr at 37°C, epidermal sheets were peeled off and washed with PBS. For dermal cell preparation, the remaining tissues following epidermal sheet removal were used. Dermal and epidermal sheets were chopped in pieces with scissors and treated with 1 mg/ml Collagenase D (Roche) and 40 µg/ml DNaseI (Sigma) for 1 hr with a magnetic stirrer. Cells were filtered with 70 µm nylon membrane and used for staining for flow cytometry.

### Statistical Analysis

Statistical analysis was performed using Graphpad Prism v4.0b software. Statistical significance was determined using non-parametric Mann-Whitney test. Differences between groups were evaluated via Tukey’s multiple comparison test. A *p-*value of <0.05 was considered significant.

## Results

### Intrathymic Medullary Accumulation of Vγ5^+^ Dendritic Epidermal T-cell Progenitors

**Figure 1:**
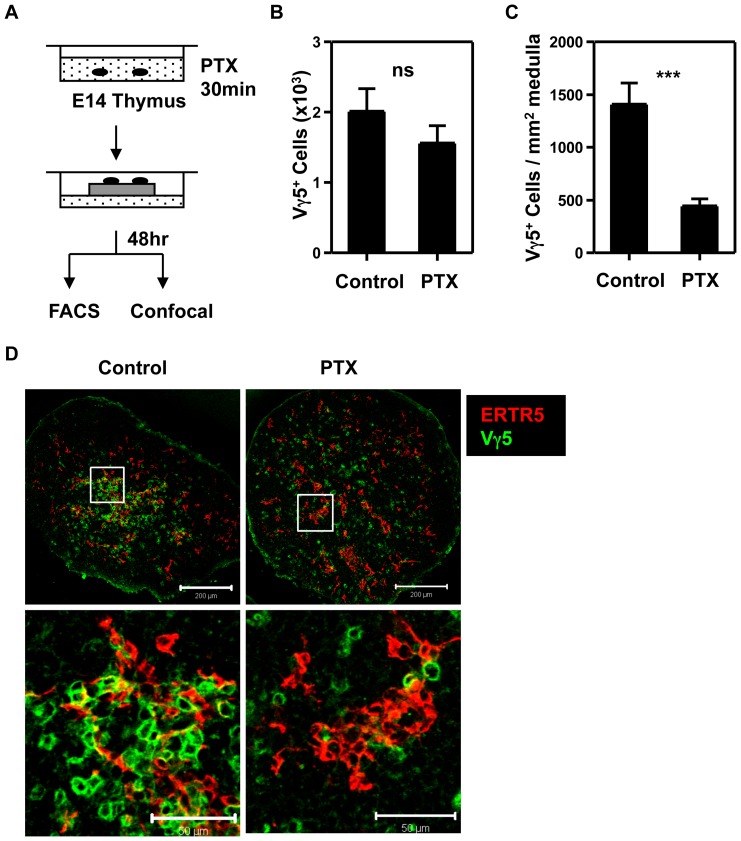
Inhibition of G-Coupled Receptor Signaling Prevents Accumulation of Vγ5^+^ DETC Progenitors in the Thymic Medulla. (A) Experimental design. Freshly isolated E14 C57BL6 thymus lobes were treated with or without 250 ng/ml Pertussis toxin (PTX) for 30 minutes, cultured as FTOC for 48 hr, then harvested for flow cytometry or confocal analysis. (B) Vγ5^+^ thymocyte numbers from PTX treated or non-treated FTOC. Control; n = 8, PTX; n = 9. (C and D) Confocal analysis of Vγ5^+^ thymocyte distribution in control and PTX treated FTOC. Error bars represents SEM, and asterisks signify a significant difference, where *p*<0.0001. Scale bars in D on upper panel represent 200 µm and on lower panel represent 50 µm.

The development of Vγ5^+^ DETC progenitors in the thymus begins during embryonic life, and involves the clustering of Vγ5^+^ thymocytes with Skint-1^+^ mTEC, an association that is evident by E17 of gestation [17]. To investigate the mechanism influencing the recruitment of Vγ5^+^ DETC progenitors to the embryonic thymic medulla, we performed short-term treatment of E14 fetal thymus organ cultures (FTOC) with Pertussis Toxin (PTX) (Figure 1A), a known inhibitor of G-protein coupled receptors, including chemokine receptors. Upon harvesting cultures 2 days after treatment, no significant difference was observed in Vγ5^+^ DETC progenitor numbers (Figure 1B) in untreated or PTX treated FTOC. In contrast, confocal quantitation to determine the anatomical distribution of Vγ5^+^ DETC progenitors in thymic sections revealed a significant inhibition of their association with ERTR5^+^ medullary areas following PTX treatment, with the majority of Vγ5^+^ DETC progenitors residing within cortical areas (Figure 1C, D). Thus, as shown previously for single positive CD4^+^ and CD8^+^ αβTCR^+^ thymocytes [23], the intrathymic migration of Vγ5^+^ DETC progenitors to thymic medullary areas is PTX sensitive.

**Figure 2:**
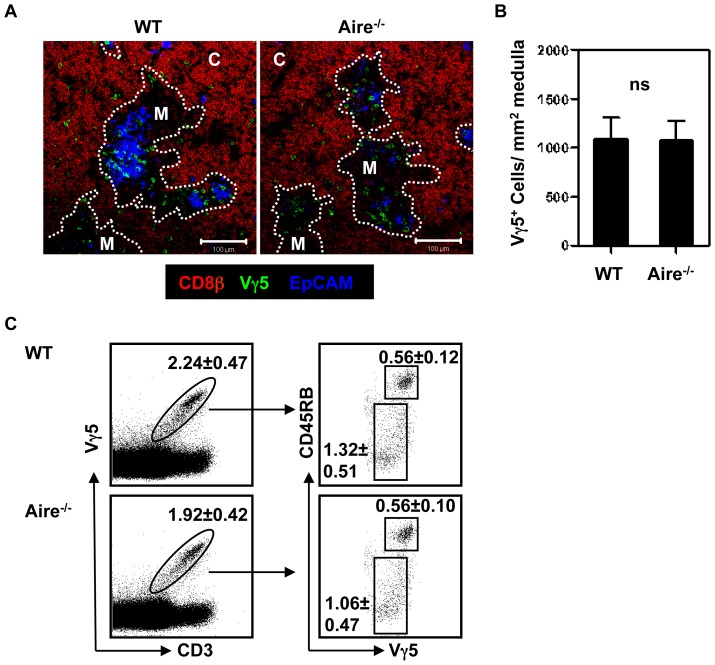
Aire is not Required for Vγ5^+^ DETC Progenitor Clustering with mTEC. (A) Representative confocal analysis of E18 WT and Aire^−/−^ thymus sections. Vγ5^+^ cells are shown in green, CD8β^+^ cells are shown in red and EpCAM^+^ medullary areas are shown in blue. M denotes medulla, C denotes cortex. Scale bars in A represent 100 µm. (B) Quantification of Vγ5^+^ cells per mm^2^ thymic medulla. WT; n = 6, Aire^−/−^; n = 6. Error bars represents SEM. (C) Individual thymus lobes of E18 WT and Aire^−/−^ embryos were teased apart and stained for Vγ5, CD3, CD24 and CD45RB expression. Numbers shown on FACS plot are the mean percentages +/− SD. WT; n = 9, Aire^−/−^; n = 4.

Our earlier studies showed that interactions with mTEC, including the Aire^+^ subset, are required for Vγ5^+^ DETC progenitor development in the thymus [17]. Recently, several reports have now linked Aire expression by mTEC to the control of intrathymic chemokine expression. For example, while the XCR1 ligand XCL1 is absent in Aire^−/−^ mice, the CCR4 ligands CCL17/CCL22, and the CCR7 ligands CCL19/CCL21 are present at reduced levels [15], [16]. Given this link between thymic chemokines and Aire, we next examined the potential link between Aire-dependent chemokines and the intrathymic migration and development of Vγ5^+^ DETC progenitors. Flow cytometric and confocal analysis of E18 WT and Aire^−/−^ thymus lobes showed no differences in the medullary accumulation of Vγ5^+^ DETC progenitors (Figure 2A, B), which were also present at normal frequencies, including immature CD45RB^low^ and mature CD45RB^high^ subsets (Figure 2C). Thus, despite being linked to alterations in thymic chemokine expression, the absence of Aire expression by mTEC does not impact upon Vγ5^+^ DETC intrathymic medullary accumulation and development.

### CCR4-Dependency of the Adult DETC Pool is not Caused by Defects in Intrathymic Development nor Neonatal Epidermal Seeding

**Figure 3:**
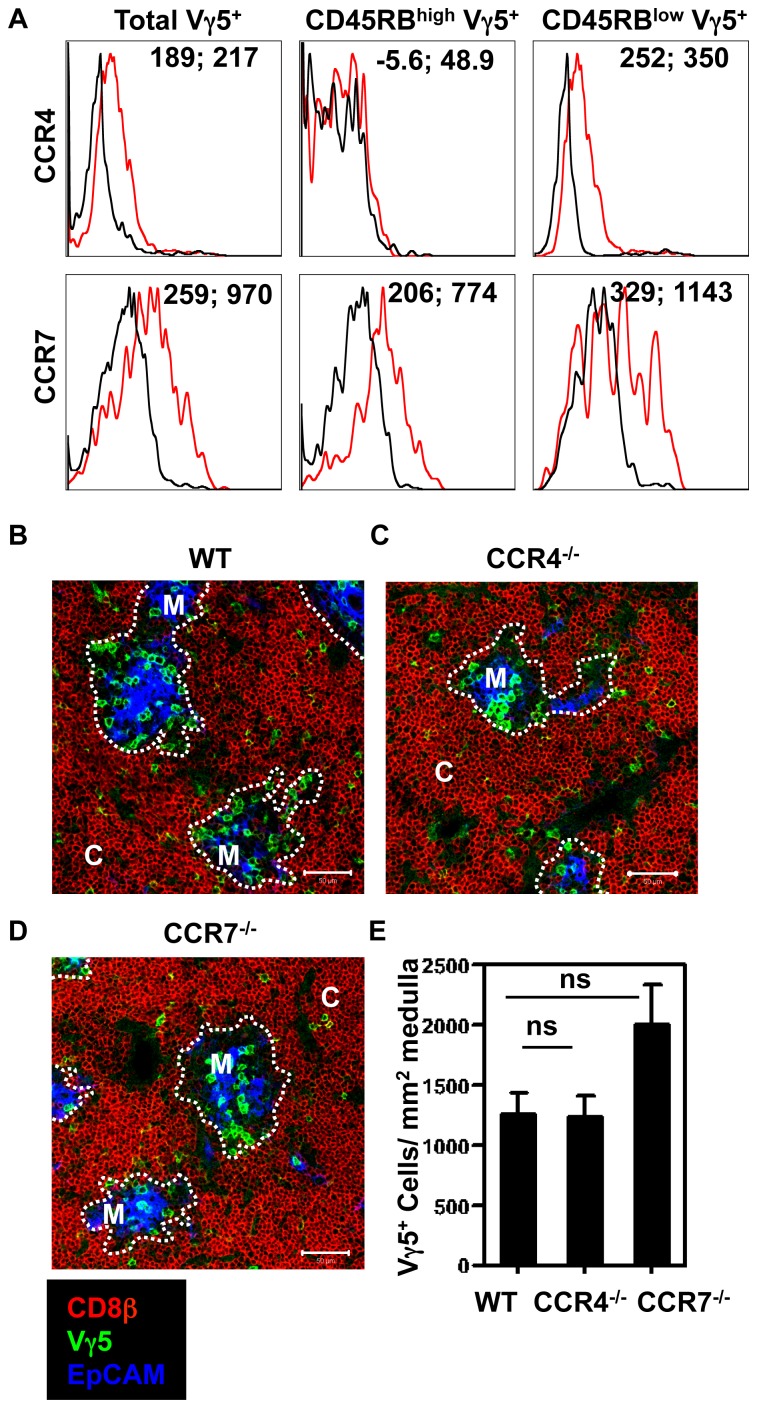
Intrathymic Medullary Accumulation of Vγ5^+^ DETC Progenitors occurs Independently of CCR4 and CCR7. (A) Expression of CCR4 and CCR7 on total Vγ5^+^ thymocytes and CD45RB^high^/CD45RB^low^ Vγ5^+^ thymocyte subsets from E18 thymus. Black histograms show the levels of antibody staining using E18 thymocytes from the indicated chemokine receptor knockout mice as a control. Red histograms show the expression level of each chemokine receptor in WT E18 thymocytes. Numbers represent the mean fluorescent intensity of CCR4/7 KO then WT cells. (B-D) Representative confocal images of E18 WT (B), CCR4^−/−^ (C), CCR7^−/−^ (D) thymus. M denotes medulla, C denotes cortex. Scale bars in B-D represent 50 µm. (E) Confocal quantification of Vγ5^+^ thymocytes per in mm^2^ medullary area in WT (n = 6), CCR4^−/−^ (n = 6) and CCR7^−/−^ (n = 6) E18 thymus. Error bars represent SEM.

**Figure 4:**
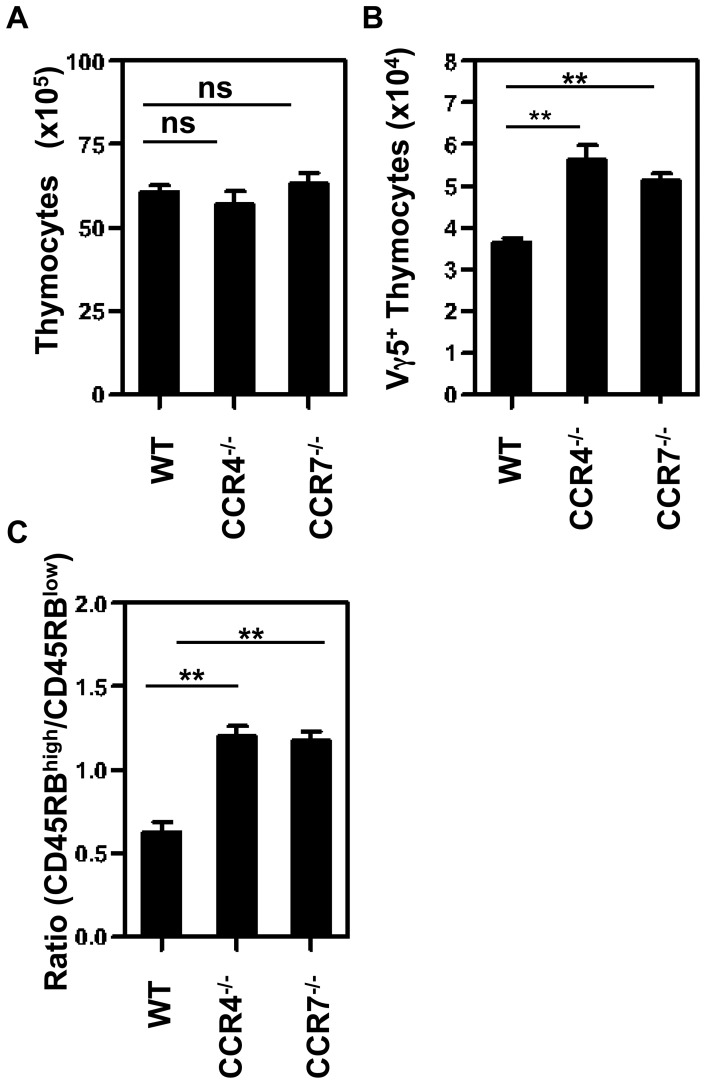
Absence of CCR4 or CCR7 causes an Increase in Mature Vγ5^+^ T Cells in E18 Fetal Thymus. (A) Total number of thymocytes from both thymus lobes of individual E18 mouse embryos of the indicated strain. (B) Total cell number of Vγ5^+^ thymocytes from E18 thymus. (C) Shows the ratio of CD45RB^high^ and CD45RB^low^ subsets within total CD3^+^ Vγ5^+^ thymocytes. A minimum of 10 mice of each strain were analyzed. Error bars represent SEM, with asterisks signifying a significant difference, where *p*<0.001.

To further define potential chemokine-chemokine receptor interactions that could be controlling the intrathymic development of Vγ5^+^ DETC progenitors, we analysed immature CD45RB^low^ and mature CD45RB^high^ subsets for their expression of a panel CCR and CXCR family members (data not shown). We focused our attention on CCR4, as it has been linked previously to DETC development [19], and was found be selectively expressed by immature CD45RB^low^ DETC progenitors (Figure 3A), and CCR7, expressed by both CD45RB^low^ and CD45RB^high^ DTEC progenitors (Figure 3A) and important in the medullary accumulation of αβT-cells [13], [30]. Analysis of thymus sections to compare the frequency of Vγ5^+^ DTEC progenitors in the thymus medulla of WT, CCR4^−/−^ and CCR7^−/−^ E18 fetal thymus revealed no significant differences (Figure 3B–E). Interestingly however, despite similar total thymocyte numbers (Figure 4A), E18 CCR4^−/−^ and CCR7^−/−^ embryos showed a slight but significant increase in Vγ5^+^ DETC thymocyte progenitor numbers compared to WT (Figure 4B), as well as a skewing of the ratio of CD45RB^low^:CD45RB^high^ cells in favour of the more mature CD45RB^high^ subset (Figure 4C).

**Figure 5:**
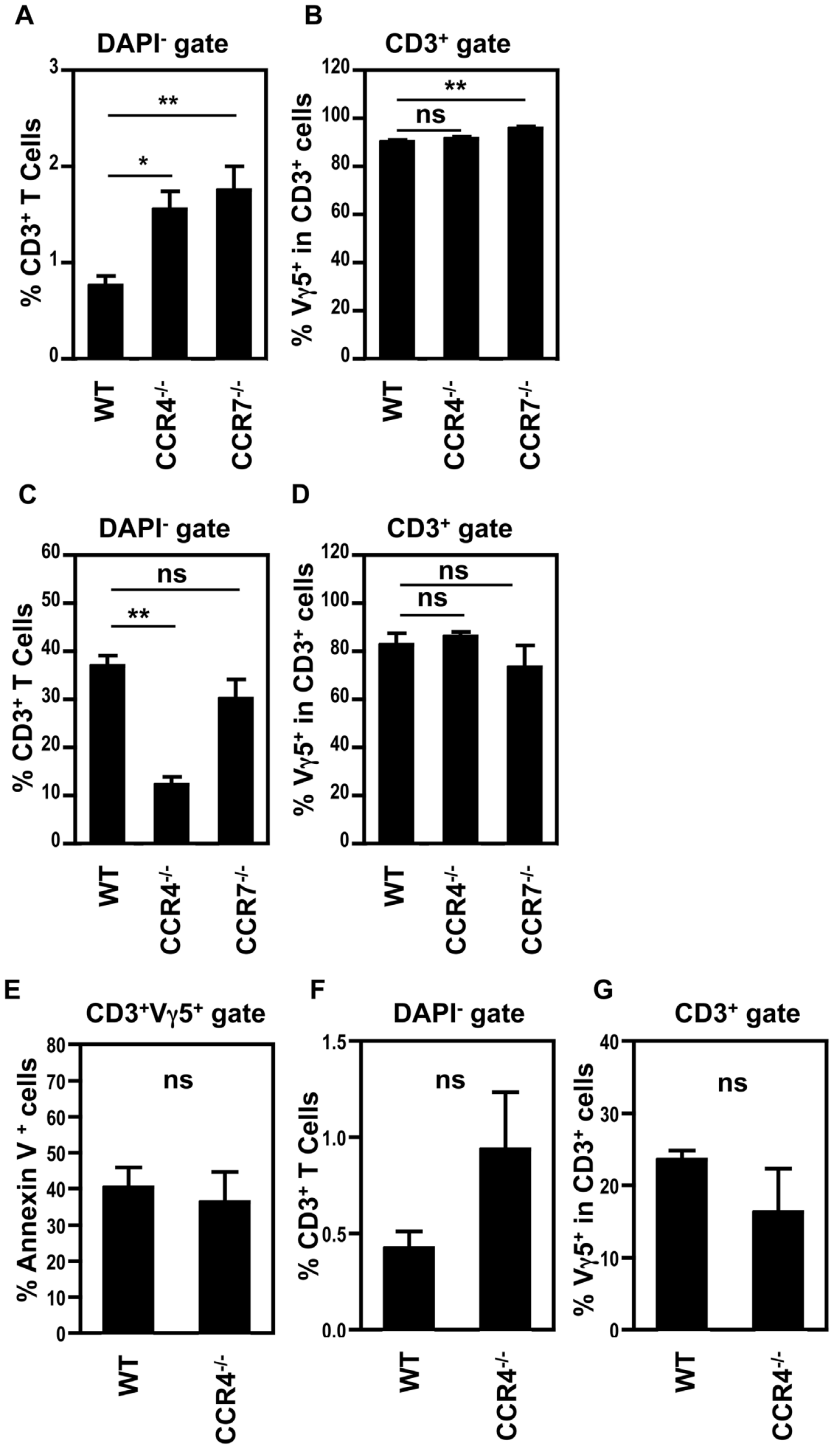
Vγ5^+^ DETC are Selectively Reduced in the Epidermis of Adult, but not Neonatal, CCR4^−/−^ Mice. (A) Day 0 newborn epidermal sheets were digested and stained for Vγ5TCR and CD3 expression. The graph shows the percentage of T-cells within DAPI- cells of the indicated strains. WT; n = 12, CCR4^−/−^; n = 8, CCR7^−/−^; n = 9. (B) Percentages of Vγ5^+^ DETC within CD3^+^ T-cells in d0 newborn epidermis. (C) Adult ear epidermal sheets were digested with Collagenase D and stained for Vγ5TCR and CD3 expression. The graph shows the percentage of CD3^+^ T-cells within DAPI- cells. WT; n = 19, CCR4^−/−^; n = 14, CCR7^−/−^; n = 12. (D) Percentage of Vγ5^+^ DETC within CD3^+^ T-cells in adult epidermis. (E) Percentage of Annexin V^+^ cells in adult ear, after gating on Vγ5^+^ DETC. WT n = 4, CCR4^−/−^ n = 3. (F) shows the percentage of T-cells in the dermis of adult ear skin in WT and CCR4^−/−^ mice, while (G) shows the proportion of Vγ5^+^ cells within dermal T cells. WT; n = 4, CCR4^−/−^; n = 4. Asterisks signify a significant difference, where ** *p*<0.001, * *p*<0.01.

While such findings are perhaps suggestive of a minor role for CCR4 and CCR7 in the thymic egress of mature CD45RB^high^ Vγ5^+^ thymocytes, no significant reduction in the proportion of Vγ5^+^ DETC was observed in either the neonatal or adult skin of CCR7^−/−^ mice, as compared to WT (Figure 5A–D). In contrast, and as reported previously [19], analysis of CCR4^−/−^ adult mice showed that while the restricted Vγ5TCR^+^ repertoire of epidermal T-cells was maintained (Figure 5D), the Vγ5^+^ DETC population was found to be significantly reduced (Figure 5C) [19]. This reduction was not due to an increased frequency of apoptotic Vγ5TCR^+^ cells (Figure 5E), nor the mis-localisation of Vγ5^+^ T-cells to the dermis of CCR4^−/−^ mice (Figure 5F, G). Interestingly, and in marked contrast to adult CCR4^−/−^ mice (Figure 5C), the Vγ5^+^ DETC compartment was not reduced in neonatal CCR4^−/−^ mice (Figure 5A, B). Collectively, these findings indicate that defects in Vγ5^+^ DETC numbers in the epidermis of adult CCR4^−/−^ mice is not due to a key role for CCR4 in either intrathymic DETC production, or initial DETC skin seeding in the neonatal period.

## Discussion

The thymic medulla plays a key role in conventional αβT-cell development, imposing both dominant and recessive central tolerance through the generation of either Foxp3^+^ regulatory T cells, or via the deletion of autoreactive T-cell clones [31]–[33]. We recently showed that the thymic medulla also provides a specialized microenvironment for the generation of a subset of invariant γδT-cells that are defined by expression of an invariant Vγ5^+^TCR, representing the progenitors of skin-homing Dendritic Epidermal T-cells (DETC) [17]. In the fetal thymus, developing Vγ5^+^ DETC progenitors accumulate within medullary regions, where they interact with mTEC expressing Skint-1. Interestingly, interactions between Vγ5^+^ DETC progenitors and mTEC includes the Aire-expressing subset of the latter, and we showed that RANKL-RANK interactions between these cells contribute to Aire^+^ mTEC development [17], [18], a process that may contribute to tolerance induction of the nascent αβT-cell repertoire [17], [34], [35]. Interestingly, Aire expression by mTEC has been shown to influence chemokine production in the thymus medulla [15], [16], a process that impacts upon the intrathymic migration of multiple cell types including dendritic cells and newly selected αβT-cells. However, despite the links between γδT-cells and Aire^+^ mTEC, the role of chemokines in intrathymic migration and development of Vγ5^+^ DETC progenitors is poorly understood.

Here, we demonstrate a role for G-protein coupled receptor mediated accumulation of Vγ5^+^ DETC precursors within embryonic thymic medulla, implicating a chemokine receptor-mediated mechanism. Despite the role of Aire in mTEC chemokine production [15], [16] and the requirement for mature mTEC during Vγ5^+^ DETC precursor maturation [17], we found normal Vγ5^+^ DETC precursor medullary accumulation in Aire^−/−^ mice. Such findings may suggest that mTEC at developmental stages prior to the Aire^+^ stage may regulate this process, which could result in the medullary attraction of Vγ5^+^ DETC precursors to stimulate mTEC maturation. Alternatively, either the production of Aire-independent chemokines by mature mTEC, or the involvement of additional cell populations other than mTEC, including medullary resident dendritic cells (DC), may explain this finding [36], [37]. In this regard, CCR7-ligands are expressed in an mTEC fraction distinct from those expressing Aire [38]. Further, medullary resident DC produce chemokines including CCL22 [39], a ligand for CCR4, a receptor shown here to be expressed by developing Vγ5^+^ DETC progenitors. Analysis of Vγ5^+^ DETC precursor clustering in CCR7^−/−^ and CCR4^−/−^ embryonic thymus did not reveal any significant defects in anatomical localization. Thus, particularly with regard to the role of CCR7, these findings highlight a differential requirement for chemokine receptor signaling in the recruitment of αβTCR^+^ thymocytes and invariant Vγ5^+^ DETC precursors to the thymic medulla.

Analysis of Vγ5^+^ DETC precursor maturation in CCR4^−/−^ and CCR7^−/−^ embryonic thymus revealed a slight, but significant intrathymic accumulation of mature CD45RB^high^ Vγ5^+^ DETC precursors, perhaps suggesting an involvement of these chemokine receptors during normal Vγ5^+^ DETC thymic emigration. Interestingly, recent evidence has also indicated that down-regulation of CCR6 during intrathymic Vγ5^+^ DETC precursor development is required for proper thymic exit, highlighting the importance of coordinated temporal expression of specific chemokine receptors [40]. Despite these intrathymic alterations, analysis of Vγ5^+^ DETC within the epidermis of CCR7^−/−^ mice at both neonatal and adult stages revealed no observable defects. In contrast, epidermal DETC were markedly reduced in adult CCR4^−/−^ mice. Importantly however, no significant alteration was found in DETC in day 0 neonatal CCR4^−/−^ mice. Our results therefore imply that initial seeding of the epidermis occurs in a CCR4-independent fashion, whilst maintenance of established DETC in later postnatal/adult phases is CCR4-dependent. Of note, while our findings on adult CCR4^−/−^ mice agrees with an earlier study [19], they contrast to the reported reduction of DETC in neonatal CCR4^−/−^ mice. The reasons for this are unclear, but given the precise age of the neonates examined was not reported, this discrepancy may reflect analysis of developmental stages subsequent to neonatal day 0 as analysed here, in which loss of DETC following normal early epidermal colonization may have already initiated. Importantly, additional studies have demonstrated a role for CCR10 in correct epidermal localization of DETC [41], providing a potential mechanism regulating the early neonatal CCR4-independent epidermal seeding. Interestingly, whilst CCR10^−/−^ mice demonstrate a reduction in DETC at neonatal stages, compensatory proliferation then partially restores adult DETC numbers, although an accumulation of DETC was reported in dermal regions was also reported. In contrast, our analysis of CCR4^−/−^ adult DETC indicated neither increased apoptosis nor aberrant dermal accumulation, while further proliferative analysis of neonatal DETC did not reveal impaired expansion within the epidermis (data not shown). Thus, the precise mechanism leading to the reduction in DETC in adult CCR4^−/−^ mice following normal neonatal epidermal seeding remains unclear, and may reflect the role of CCR4 in DETC retention as opposed to epidermal colonization.

Overall, we report that the medullary localization of Vγ5^+^ DETC precursors in fetal thymus, while important for both Vγ5^+^ DETC precursor maturation and Aire^+^ mTEC induction, occurs in the absence of both Aire-mediated effects on chemokine expression by mTEC and CCR4 and CCR7 mediated migration of Vγ5^+^ DETC progenitors. Further, we report that whilst initial seeding of DETC occurs in a CCR4-independent manner, conversely maintenance of the established epidermal DETC compartment in the adult requires CCR4, collectively indicating a differential role of chemokine-mediated medullary attraction and retention between conventional diverse αβT-cells and innate-like invariant Vγ5^+^ DETC precursors.
